# Characterization of a transcriptional TPP riboswitch in the human pathogen *Neisseria meningitidis*

**DOI:** 10.1080/15476286.2020.1727188

**Published:** 2020-02-20

**Authors:** Francesco Righetti, Solange Lise Materne, John Boss, Hannes Eichner, Emmanuelle Charpentier, Edmund Loh

**Affiliations:** a Department of Microbiology, Tumor- and Cell Biology, BioClinicum, Karolinska University Hospital, Stockholm, Sweden; b Max Planck Unit for the Science of Pathogens, Berlin, Germany; c Department of Regulation in Infection Biology, Max Planck Institute for Infection Biology, Berlin, Germany; d Institute for Biology, Humboldt University, Berlin, Germany; e Department of Regulation in Infection Biology, Helmholtz Centre for Infection Research, Braunschweig, Germany; f The Laboratory for Molecular Infection Medicine Sweden (MIMS), Umeå Centre for Microbial Research (UCMR), Department of Molecular Biology, Umeå University, Umeå, Sweden; g SCELSE, Nanyang Technological University, Singapore, Singapore

**Keywords:** *Neisseria meningitidis*, riboswitch, RNA, gene regulation, TPP, vitamin, regulatory RNA, non-coding RNA, pathogen

## Abstract

Increasing evidence has demonstrated that regulatory RNA elements such as riboswitches (RS) play a pivotal role in the fine-tuning of bacterial gene expression. In this study, we investigated and characterized a novel transcriptional thiamine pyrophosphate (TPP) RS in the obligate human pathogen *N. meningitidis* MC58 (serogroup B). This RS is located in the 5´ untranslated region upstream of *thiC* gene, encoding a protein involved in TPP biosynthesis, an essential cofactor for all living beings. Primer extension revealed the transcriptional start site of *thiC*. Northern blot analysis of *thiC* mRNA and reporter gene studies confirmed the presence of an active TPP-sensing RS. Expression patterns of the wild-type RS and site-specific mutants showed that it is an OFF switch that controls transcription elongation of *thiC* mRNA. Interestingly, the regulatory mechanism of the meningococcal *thiC* RS resembles the Gram-positive *Bacillus subtilis thiC* RS rather than the Gram-negative *Escherichia coli thiC* RS. Therefore, the meningococcal *thiC* RS represents a rare example of transcriptional RS in a Gram-negative bacterium. We further observed that the RS is actively involved in modulating gene expression in response to different growth media and to supplemented bacterial and eukaryotic cell lysates as possible sources of nutrients in the nasopharynx. Our results suggest that RS-mediated gene regulation could influence meningococcal fitness, through the fine-tuning of biosynthesis and scavenging of nutrients and cofactors, such as thiamine.

## Introduction

Riboswitches (RSs) are *cis*-acting regulatory RNA elements often found in bacteria that are capable of living in multiple different environments, such as *Bacillus subtilis* and *Escherichia coli* [1,2]. RSs enable these bacteria to modify their gene expression by sensing the concentration of specific ligand molecules/metabolites in their proximity. A typical RS consists of two domains, the aptamer and the expression platform, and is located within the 5´ untranslated region (UTR) of genes involved in production/uptake of the metabolite it senses. Upon binding of the metabolite to the aptamer, a conformational change affects downstream gene expression, by either promoting or blocking transcription elongation or translation or affecting mRNA stability. A variety of inorganic (ions) and organic (vitamins, nucleotides, amino acids and other metabolites) compounds can be recognized by specific RNA architectures.

A well characterized example is the thiamine pyrophosphate (TPP, activated vitamin B1) RS [3,4]. The TPP-binding aptamer structure has been characterized at atomic resolution and investigated with a variety of biophysical and biochemical methods [5–14]. TPP RSs typically control the expression of genes involved in TPP biosynthesis or the transport of its precursor molecules. TPP is a coenzyme of several enzymes involved in the utilization of carbohydrates as an energy source and therefore plays a pivotal role in the central metabolism and the survival of the bacterium.

The discovery of novel RSs and the screening of sequenced genomes rely largely on computational approaches based on sequence and structure conservation of the known aptamer motifs. Despite the accuracy achieved by the computational models, the experimental validation of the ligand-based response and the determination of the regulatory mechanism involved are necessary.

*Neisseria meningitidis* is a Gram-negative diplococcal obligate human pathogen, responsible for causing fatal infections such as acute meningitis and septicaemia [15,16]. Unlike many bacterial species able to grow in different environments, *N. meningitidis* colonizes exclusively the epithelial surface of the nasopharynx with no other known habitat. Upon entry into the blood circulation, *N. meningitidis* can proliferate causing septicaemic shock and/or penetrate the blood-brain barrier inducing acute meningitis. Other important bacterial pathogens, such as *Streptococcus pneumoniae* and *Haemophilus influenzae*, also reside on the same mucosal surface of the nasopharyngeal cavity and are able to cause similar infections such as meningitis and septicaemia [17,18].

Recent investigations on human commensal microbial communities highlighted the importance of the dynamic relationships between bacterial species and with the host environment [19]. In particular, the commensal microflora is competing for nutrients, such as carbon and nitrogen sources, metal ions and vitamins. In addition, to successfully colonize the human host, bacteria also have to elude the host immune system and survive eventual activation of its defence mechanisms. However, to date little is known about the adaptation processes of nasopharyngeal bacteria in this distinctive niche, especially their underlying genetic regulations.

Even though *Neisseria* is known to widely use regulatory RNA elements to control gene expression, no RS has been investigated to date [20–25]. Here, we identified four RS candidates in *N. meningitidis* serogroup B (MC58) genome, two of which are classified as TPP elements. In this study, we characterized an uncommon transcriptional TPP RS in *N. meningitidis* that controls the expression of *thiC*, coding for an enzyme involved in the biosynthesis of TPP. The human nasopharynx where *N. meningitidis* colonizes is considered to be lacking crucial nutrients for its survival. Our findings here suggest that *N. meningitidis* utilizes the TPP RS regulation to balance the synthesis and scavenge of thiamine to facilitate its survival. In addition, using Riboswitch Scanner, we identified four and three putative TPP RSs candidates in *S. pneumoniae* and *H. influenzae*, respectively. The presence of TPP RSs might suggest a common sensing and scavenging mechanism among these pathogens living in the nasopharynx.

## Results

### Identification of meningococcal riboswitch candidates

To identify putative meningococcal RS elements, the genome sequence of *N. meningitidis* MC58 chromosome (accession number: NC_003112.2) was computationally analysed using ‘Riboswitch Scanner’ [26]. The programme identified four putative elements (Table S1). A preQ1 RS candidate was located upstream NMB0317 (*queF*), coding for a 7-cyano-7-deazaguanine reductase; an S-adenosylmethionine (SAM) RS mapped upstream NMB1799 (*metK*), coding for an S-adenosylmethionine synthetase; two TPP RSs were identified upstream NMB2040 (*thiC*) and NMB2067 (*cytX*), the first ORF of the polycistronic operon that comprises also *thiO* and *thiE*. ThiC is an enzyme that catalyses the conversion of aminoimidazole ribotide to hydroxymethylpyrimidil phosphate (HMP-P), an early reaction in the biosynthesis of TPP. CytX is a transporter that imports hydroxymethylpyrimidine (HMP). ThiO and ThiE are the other two enzymes involved in the synthesis of TPP (see Fig. S1).

**Figure 1.**
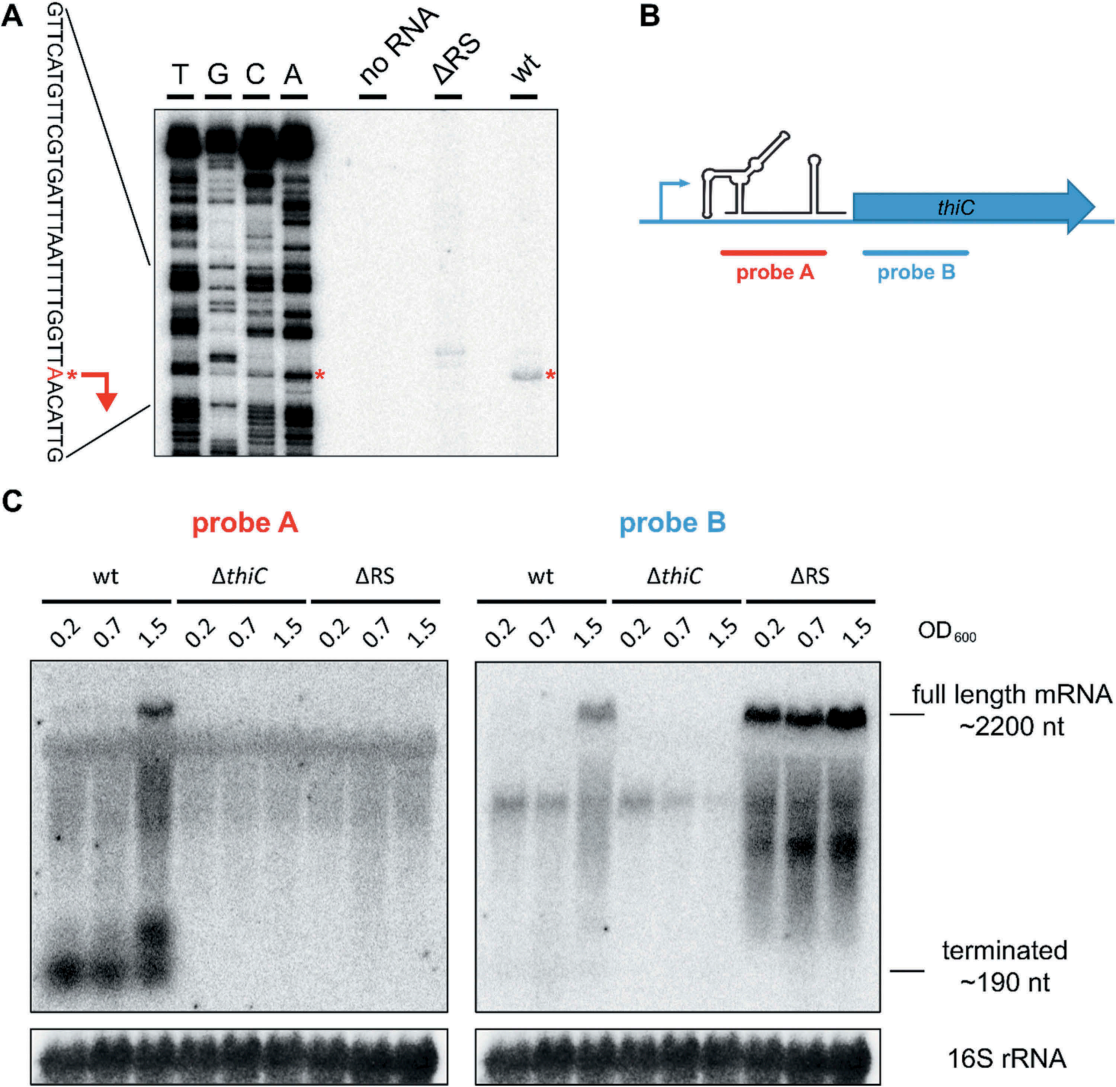
Primer extension and northern blot analysis of *thiC* mRNA during meningococcal growth. (A) In order to identify the transcriptional start site of *thiC* (marked with red asterisks and arrow), primer extension reaction was performed with RNA isolated from *N. meningitidis* MC58 (wt) and ΔRS mutant, in which only the RS region upstream *thiC* is removed and therefore lacking the region where the radiolabelled primer anneals. ‘No RNA’ is the control reaction without RNA. (B) A schematic figure of the neisserial *thiC* gene. The location of both Northern blot probes was designed to anneal either to the *thiC* 5´UTR (probe A, red) or to the protein-coding region (probe B, blue). (C) RNA was isolated from *N. meningitidis* MC58 wt, a *thiC* (including the promoter and 5´UTR) knock out mutant (Δ*thiC*) and a ΔRS mutant. Northern blot detection of *thiC* mRNA with probe A shows two RNA transcripts. A short fragment of about 190 nt is highly abundant at low OD_600_ and the signal intensity decreases with the growth. The full-length mRNA (2200 nt) signal appears only at high OD_600_. The short fragment is not detected with probe B, indicating that it corresponds to the terminated 5´UTR. As expected, the mRNA is not present in the Δ*thiC* strain. In the case of ΔRS strain, probe B identifies the full-length mRNA, and its expression is independent of the growth phase.

Previous extensive investigations on the regulation of genes involved in thiamine synthesis highlighted *thiC* as the gene most frequently controlled by TPP RSs among bacterial genomes [27]. Visual analysis of the genomic region upstream *thiC* revealed a conserved promoter comprising −35 and −10 regions. The transcriptional start site was identified by primer extension, mapping a 193 nt long *thiC* 5´UTR (Fig. 1(A)). In this analysis, we included RNA from a ΔRS strain, which harbours a deletion of the predicted TPP RS region within the 5´UTR of *thiC*. This control was included to verify the specificity of the primer used for the primer extension reaction.

### *Validation and characterization of the neisserial* thiC *TPP riboswitch*

The terminal part of the *thiC* 5´UTR presents a stable stem-loop structure, followed by a stretch of seven uridines (Fig. 3(A)). This configuration resembles a typical Rho-independent transcriptional terminator, often present in the expression platform of transcriptional RSs. Following this hint, Northern blot analysis was performed on total RNA isolated from meningococcal cultures harvested at different growth stages (Fig. 1). Two probes were designed to anneal to the 5´UTR (probe A) and to the protein-coding region (probe B) (Fig. 1(B)) of *thiC*. Northern blot detection of *thiC* mRNA with probe A shows two species (Fig. 1(C)). The short fragment of about 190 nt is highly abundant at low OD_600_ and the signal intensity decreases with the growth. Concomitantly, the intensity of the full length (about 2200 nt) mRNA signal increases. The short fragment is not detected with probe B, indicating that it corresponds to the terminated 5´UTR.

Two additional mutant strains were constructed and included in the analysis. A Δ*thiC* mutant harbouring a full deletion of the *thiC* locus, including the promoter, 5´UTR and protein-coding region up to the translational stop codon. In the ΔRS strain, only the region comprising the aptamer of the *thiC* RS and part of the terminator stem-loop was deleted, leaving its intact native promoter and ribosome binding site (RBS). Both Δ*thiC* and ΔRS strains did not show a growth defect when cultured in rich medium. As expected, *thiC* mRNA is not expressed in the Δ*thiC* strain (Fig. 1(C)). In the case of the ΔRS strain, probe B identifies only the full-length mRNA, and its expression is independent of the growth phase (Fig. 1(C)).

These findings support the hypothesis that *thiC* regulation in response to growth phase is mediated by an RNA element located within the 5´UTR, rather than by transcription initiation controlled by the promoter.

**Figure 2.**
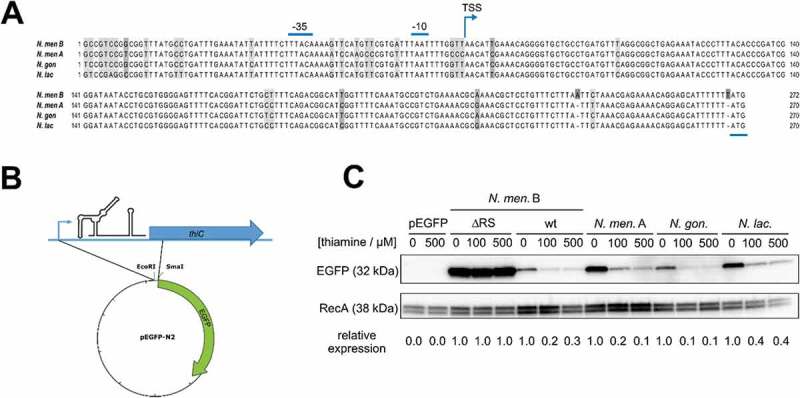
Ectopic reporter gene study of the neisserial *thiC* RS. (A) The promoter region (−35 and −10 elements) and the 5´UTR are conserved within the different *Neisseria* species. The transcriptional start site (TSS) is indicated with a blue arrow and the translational start codon (ATG) underlined in blue. (B) A schematic figure of the neisserial TPP RS-EGFP fusion plasmid. The genomic region corresponding to the promoter, 5´UTR and ATG start codon of *thiC* from *N. meningitidis* serogroups B and A, *N. gonorrhoeae* and *N. lactamica* were cloned in a translational fusion with EGFP. (C) The expression of EGFP in *E. coli* was analysed by Western blot, in the absence and increasing concentration of thiamine. In ΔRS background (deletion of RS region), the expression of EGFP was constitutively high regardless of additional thiamine, while the other neisserial RS clones showed thiamine-dependent EGFP expression. Empty plasmid (pEGFP), harbouring no *thiC* promoter and its 5´UTR was used as control. EGFP signals were quantified and normalized to the corresponding RecA signals and each set by the value of the 0 μM thiamine sample.

The nucleotide sequences of the *thiC* promoter region and its 5´UTR are highly conserved among different *Neisseria* species (Fig. 2(A)). In order to validate the functionality of this putative TPP RS, ectopic reporter gene studies were set up. The genomic region corresponding to the promoter, 5´UTR and ATG start codon of *thiC* from *N. meningitidis* serogroups B and A, *N. gonorrhoeae* and *N. lactamica* were cloned in a translational fusion in a plasmid encoding EGFP (Fig. 2(B)). The same region amplified from the ΔRS strain was also cloned. The resulting plasmids were transformed into *E. coli* and the obtained strains were grown in Luria-Bertani (LB) medium or in LB supplemented with increasing concentrations of thiamine. Western blot analysis of the total protein lysates shows that the EGFP amount decreases with increasing concentration of supplemented thiamine and this regulation is dependent on the element encoded within the cloned 5´UTR (Fig. 2(C)). Moreover, the region cloned from each *Neisseria* strain confers a comparable thiamine-dependent regulation, suggesting that the TPP RS functionality is broadly conserved among different *Neisseria* species.

### *Analysis of the structure and mechanism of the* thiC *riboswitch*

**Figure 3.**
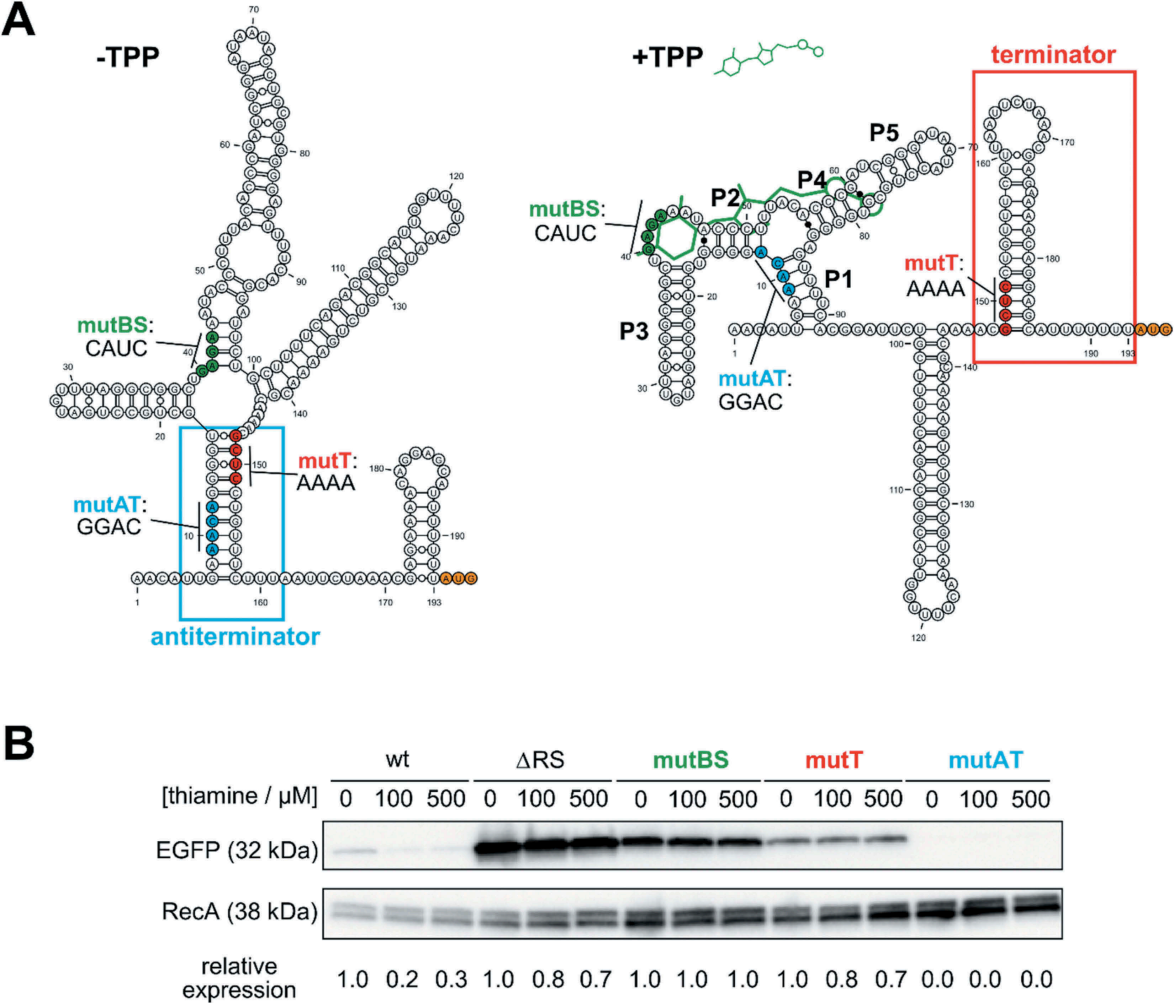
Structural and functional analysis of *thiC* RS. (A) The secondary structure of the *thiC* 5´UTR up to translational start codon (AUG, in orange) was computationally predicted. The conformational change induced by the binding to TPP (schematized in green) disrupts the antiterminator structure (in blue) and promotes the formation of the terminator element (in red). The site-directed mutations affecting the TPP-binding site (mutBS), the antiterminator (mutAT) and the terminator (mutT) are indicated in green, blue and red, respectively. (B) The ectopic expression of EGFP fused to the different mutated *thiC* 5´UTR was analysed by Western blot, in the absence and various concentrations of thiamine. ΔRS, mutBS and mutT showed high expression of EGFP regarding additional thiamine. mutAT showed no expression of EGFP. The wild-type (wt) clone was used as control. EGFP signals were quantified and normalized to the corresponding RecA signals and each set by the value of the 0 μM thiamine sample.

To further characterize the mechanism of this *thiC* RS, the RNA secondary structure of the 5´UTR was investigated. Based on the previously well characterized TPP RSs from *E. coli* and *B. subtilis*, the two alternative folding architectures of *thiC* RS were modelled (Fig. 3(A), see also Fig. S2) [27,28]. Notably, the proposed configurations support a Rho-independent transcriptional termination mechanism, similar to what was observed in the Gram-positive *B. subtilis* TPP RSs [3]. In contrast, RSs in Gram-negative bacteria often exert their function on the translational level, such as *E. coli thiM* TPP RS [4,8,9]. In addition, the *E. coli thiC* TPP RS could also couple Rho-dependent transcriptional termination and translational inhibition [29,30].

The regulatory mechanism proposed for the neisserial thiC RS is based on two mutually exclusive structures within the expression platform: an antiterminator and a Rho-independent terminator. In absence of the ligand TPP, the antitermination stem can form and this configuration blocks the 5´ branch of the terminator stem. Therefore, the RNA polymerase will produce the full-length mRNA. In contrast, when TPP is present and binds to the nascent RNA molecule, a shift in the P1 stem liberates the 5´ branch of the terminator allowing the termination structure to fold. In this state, the RNA polymerase will abort transcription immediately upstream the translation initiation codon.

To validate the proposed mechanism, a series of site-directed mutations were designed to compromise these key structural elements (Fig. 3(A)). A stretch of four highly conserved nucleotides (GAGA) known to be involved in the binding of the ligand were changed (CAUC), aiming to disrupt the TPP-binding site (mutBS) [9,12]. A mutation of four nucleotides (GCUC to AAAA) of the terminator (mutT) is predicted to significantly weaken the stem because it breaks three G-C pairings at the base of the stem (minimum free energy of the optimal secondary structure calculated with RNAfold: −5.30 kcal/mol; the wild-type stem-loop is −14.90 kcal/mol). The third mutation aims to impair the stability of the antitermination structure (mutAT) (AACA to GGAC), without impacting the binding of TPP.

These mutated 5´UTRs were cloned in the translational reporter fusion system and introduced by transformation into *E. coli* as previously described. The Western blot analysis shows that both mutations that affect the binding site and the terminator cause impairment in the TPP-dependent regulation of EGFP expression, with constant EGFP expression regardless of TPP concentrations (Fig. 3(B)). This result also confirms that the mutBS 5´UTR is unable to sense TPP and the mutT 5´UTR cannot prematurely terminate the mRNA synthesis. The level of EGFP produced by the mutAT strain could not be detected at any concentration of supplemented thiamine (Fig. 3(B)). This is in agreement with the induced destabilization of the antitermination structure and consequent favouring of the terminator configuration.

### *The RS-mediated expression of* thiC *is affected by different culturing media and bacterial or cell lysates*

**Figure 4.**
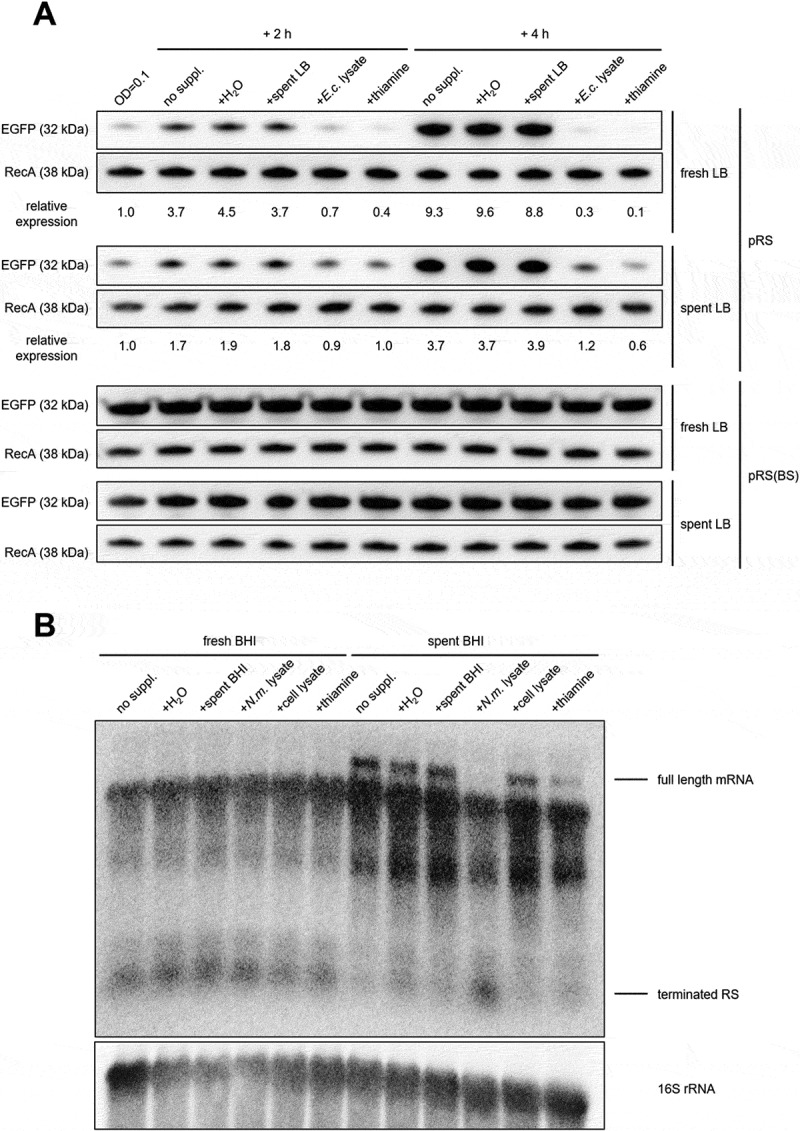
Modulation of *thiC* expression in different growth media and bacterial or cell lysates. (A) *E. coli* carrying the reporter plasmid with EGFP fused to the wild-type *N. meningitidis thiC* promoter plus 5´UTR (pRS) or the variant with the mutation in the TPP binding region (pRS(BS)) was cultured in fresh or spent LB up to OD_600_ = 0.1 and supplemented with H_2_O, spent LB, *E. coli* lysate and pure thiamine. After 2 and 4 h, bacterial samples were harvested and analysed by Western blot. EGFP signals were quantified and normalized to the corresponding RecA signals and each set by the value of the OD = 0.1 sample. (B) *N. meningitidis* was grown in either fresh or spent BHI supplemented with H_2_O, spent BHI, *N. meningitidis* lysate, A459 human alveolar epithelial cell lysate, and pure thiamine. Northern blot analysis was performed using probe A from Fig. 1(B).

A precise regulation of biosynthetic pathways is a key feature to maximize the fitness of bacteria that reside in nutritionally poor environments as well as facing competition with other commensal organisms. We, therefore, tested the behaviour of *thiC* RS when bacteria are exposed to different culturing media with and without the addition of thiamine and bacteria/cell lysates (Fig. 4).

First, we monitored the *thiC* RS-mediated expression of EGFP, when the *E. coli* reporter strain was grown in fresh and spent LB medium, with the addition of thiamine sources as pure solution or *E. coli* lysate (Fig. 4(A)). The spent LB medium, used as a nutrient-depleted medium, was obtained by growing *E. coli* in fresh LB medium up to stationary growth phase and removing bacteria by filtration (see material and methods). *E. coli* cells were lysed mechanically, and the supernatant was used as a nutrient-rich supplement. Results confirm a progressive increase of EGFP levels over time due to reduction of available thiamine in the medium. This effect could be reverted by addition of both pure thiamine and *E. coli* lysate, indicating that *E. coli* can promptly uptake thiamine sources from the medium and the RS can then quickly block the expression of EGFP. This repressive effect is visible also in the case of growth in spent LB, although less pronounced as expected. The different growth media, growth phases and supplements had no effect on the expression of EGFP when the plasmid contained the RS with mutated binding site (pRS(BS)), confirming that the regulatory effect is entirely dependent on a fully functional TPP RS.

To further validate the TPP RS effect in its native background, we analysed the *thiC* mRNA expression profile in *N. meningitidis* using a similar experimental setup (Fig. 4(B)). Total RNA was isolated from meningococcus grown in both fresh and spent BHI until early exponential phase. Northern blot showed that the transcription of the full-length *thiC* mRNA is only activated in spent medium (Fig. 4(B)). Supplementing the spent medium with meningococcal lysate induced a clear premature transcriptional termination. However, the addition of thiamine caused partial reduction of the full-length *thiC* mRNA and a non-detectable increase of the terminated fragment (Fig. 4(B)). Interestingly, supplementing with human alveolar epithelial cells (A459) lysate did not induce a premature transcription termination.

### *Analysis of the second neisserial putative TPP riboswitch and* thiD *5´UTR*

**Figure 5.**
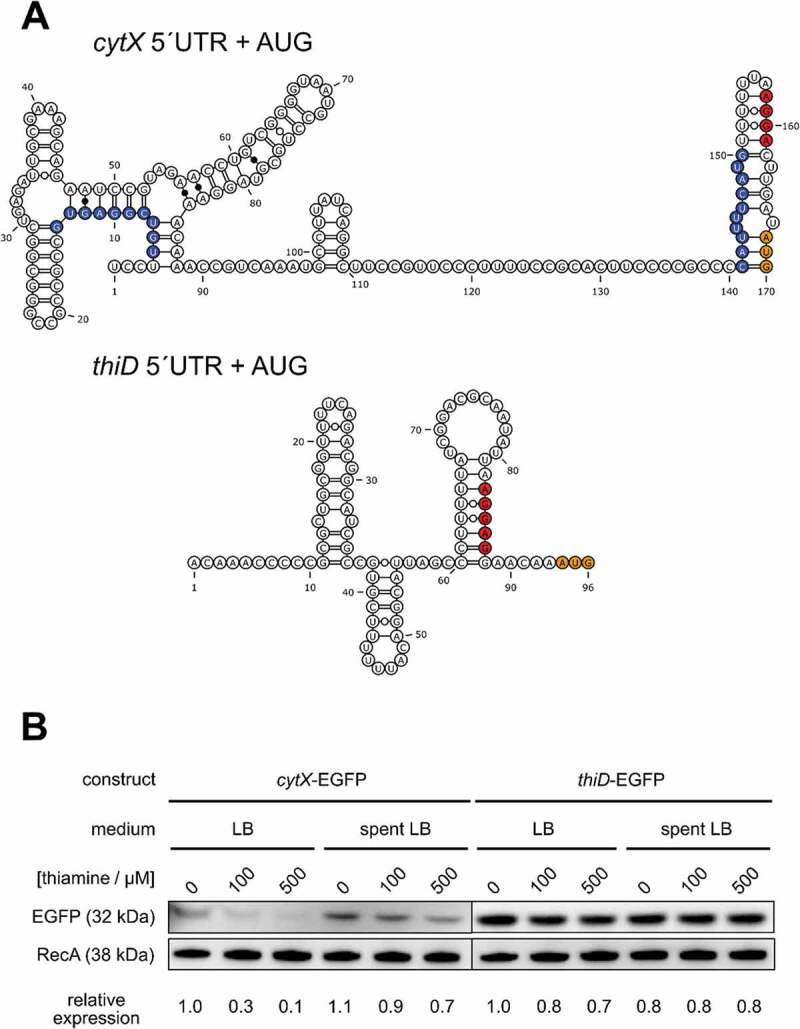
Structural analysis and ectopic reporter gene study of the *cytX* and *thiD* 5´UTR. (A) The predicted secondary structures of *cytX* TPP RS and *thiD* 5´UTRs (with RNAfold) are displayed. RBS and translational start codon (AUG) are depicted in red and orange, respectively. The alternative structure that could liberate the RBS of *cytX* while TPP is scarce is highlighted in blue. (B) The region corresponding to the promoter, 5´UTR and first 15 coding bp of *cytX* or 30 coding bp of *thiD* from *N. meningitidis* serogroups B were cloned in a translational fusion with EGFP. The expression of EGFP in *E. coli* was analysed by Western blot, in full (LB) and spent rich medium (spent LB), supplemented with thiamine. EGFP signals were quantified and normalized to the corresponding RecA signals and each set by the value of the LB 0 μM thiamine sample.

The second TPP riboswitch candidate identified by Riboswitch Scanner is located within the 5´UTR of *cytX* (NMB2067), coding for the HMP transporter. KEGG genome data of *N. meningitidis* MC58 suggest that *cytX* is cotranscribed with the downstream genes *thiO* and *thiE*, coding for two enzymes involved in TPP biosynthesis [31]. An RS controlling *cytX* could, therefore, affect the expression of *thiO* and *thiE*. A predicted secondary structure of *cytX* TPP RS is shown in Fig. 5(A).

The upstream region of *cytX* corresponding to its putative promoter, 5´UTR (167 bp) and first 15 coding bp of *cytX* was cloned in the EGFP reporter plasmid and tested in *E. coli* for thiamine-dependent regulation (Fig. 5). Addition of thiamine causes repression of EGFP expression in LB and the effect is more pronounced in spent medium, comparable to the regulation previously observed in the case of *thiC* RS. We further investigated the upstream region of another gene involved in TPP biosynthesis, the *thiD* gene (NMB1616). ThiD is a kinase, central in the pathway but its 5´UTR does not seem to contain a TPP RS based on sequence and secondary structure analyses (Fig. 5(A)). Reporter gene study with fusion of the putative promoter, 5´UTR (93 bp) and its first 30 coding bp confirms that the *thiD* upstream region does not confer thiamine-dependent regulation and therefore lacks an RS element (Fig. 5).

## Discussion

In this work, we characterized the first riboswitch in *N. meningitidis*. The neisserial *thiC* TPP RS is a transcriptional switch that upon binding to TPP effectively terminates transcription upstream of the protein-coding region. Previous publications based on experimental evidences and structural predictions show that TPP RSs in Gram-negative bacteria make use of almost exclusively translational mechanisms. In light of this, the meningococcal *thiC* RS represents a notable exception. Our results suggest that the configuration of the expression platform and the regulatory mechanism of *thiC* in *Neisseria* resemble those of typical TPP riboswitches in Gram-positive species, such as the *B. subtilis thiC* RS (Fig. S2). Both of these TPP RS structures are able to form a stable Rho-independent transcriptional terminator directly upstream the coding region. It is unclear why an organism or a bacterial species group utilizes prevalently a specific regulatory mechanism over the others. It has been suggested that transcriptional RSs offer a the cost-effective advantage derived from not synthesizing the mRNA molecule when not necessary. It is speculated that transcriptional RSs are therefore preferred in genetic configurations where genes are organized in long polycistronic operons, which are more common in Gram-positive bacteria [32]. In this scenario, a single RS can regulate a cluster of genes simultaneously and efficiently. However, the neisserial *thiC* is transcribed as a monocistronic unit.

In *E. coli*, three TPP RS elements are reported and are located upstream of individual genes: *thiC, thiM* and *thiB* [4,29,30,33]. These genes are the first of their respective polycistronic operons: *thiCEFSGH, thiMD, thiBPQ*, that comprises all the genes essential for TPP biosynthesis and related transporters. Although all three RS have been predicted to block translation via sequestration of the RBS region, experimental evidences have highlighted potentially different mechanisms. Initial studies of *thiM* and *thiC* RSs solved the mutually exclusive structures that sequester or liberate the RBS upon binding to TPP and therefore regulate translation [4]. Recent works on *E. coli thiC* RS showed that binding of TPP affects transcriptional pausing and ultimately induces Rho-dependent premature transcriptional termination [29,30,33]. In this case, the regulatory mechanism involves both transcription termination and translation repression. A TPP-dependent transcriptional pause site was also identified in the *E. coli thiB* RS, but not in its *thiM* RS [30]. The effect of cotranscriptional binding of the ligand to the aptamer on transcriptional pausing, mRNA folding and downstream gene(s) expression might have been overlooked. In contrast, the five TPP RSs identified in *B. subtilis* all seem to regulate transcription elongation of the downstream genes, including *thiC* (Fig. S2) [27].

The second putative TPP RS predicted in *N. meningitidis* is located upstream of *cytX*, potentially cotranscribed with the downstream genes *thiO* and *thiE*. Using an *E. coli* reporter system, we observed thiamine-dependent regulation mediated by *cytX* 5´UTR, supporting the RS prediction (Fig. 5(B)). This element does not present any obvious transcriptional terminator structure but can potentially hinder the RBS and the proposed mechanism is therefore translational (see Fig. 5(A)). Moreover, the predicted architecture of the expression platform of the neisserial *cytX* RS resembles that of the *E. coli thiC* one, suggesting a possible involvement of transcriptional pauses and Rho-dependent transcriptional termination. Interestingly, unlike in many bacterial species, the neisserial *thiD* gene (encoding the phosphomethylpyrimidine kinase that phosphorylates HMP imported by CytX or HMP-P produced by ThiC) lacks a TPP RS and is constitutively expressed, regardless of thiamine concentration, as observed in our reporter gene studies (Fig. 5(B)). Conservation of RS-mediated regulation in at least some of the genes involved in thiamine uptake and production suggests that precise control is beneficial for bacterial fitness. How *Neisseria* regulates the expression of the other genes involved in thiamine metabolism remains to be elucidated.

A recently developed computational pipeline has been used to identify novel classes of ncRNA candidates in several bacterial genomes [34]. Among the 30 ncRNA candidates discovered, a putative RS was found associated with genes involved in the biosynthesis of TPP, in particular with *thiS* (encoding for a sulphur carrier protein involved in the synthesis of thiamine), and therefore termed *thiS* motif. This *thiS* motif is conserved in actinobacteria, firmicutes and metagenomic DNA datasets. A deeper characterization of this RS unveiled that the aptamer selectively binds HMP, an intermediate in the biosynthesis of thiamine, instead of TPP [35]. An *in silico* investigation suggests that this *thiS* motif is not present in *Neisseria*. However, the *thiS* RS discovery opens to the possibility that other RNA architecture sensing intermediate molecules could be involved in regulating genes of the thiamine biosynthetic pathway.

Thiamine is one of the crucial cofactors for cellular wellbeing of all organisms. Thiamine can only be produced in bacteria, fungi and plants, but not in mammals. Therefore, humans must obtain thiamine through dietary intake. A publication has shown that intestinal microbiota is also an important source of thiamine and can be absorbed into the intestinal epithelium via their thiamine transporters [36]. In addition, non-thiamine producing bacteria residing in the human gut would also utilize thiamine produced by other bacteria as well as from the host diet for their own need [19,36]. Unlike the human gut, the nasopharynx is food free thus is considered to contain less available thiamine. This suggests that nasopharyngeal bacteria such as *N. meningitidis* would need to either produce their own thiamine or scavenge it from other thiamine-producing bacteria. Indeed, we observed that a deletion of the *cytX-thiOE* entire operon, in a Δ*thiC* background of *N. meningitidis* is not viable even if cultured in rich medium (data not shown). This observation further indicates that a functional biosynthetic TPP pathway and uptake are of key importance for its fitness. In *Neisseria*, we showed that *thiC* TPP RS is triggered not only during the adaptation to nutrient-depleted medium (to activate thiamine production) but also when spent medium is supplemented with sources of thiamine or its precursors (to repress thiamine production) (Fig. 4). Interestingly, human epithelial cell lysate supplement does not affect the expression of *thiC* mRNA, perhaps because human cells contain a very limited amount of thiamine and precursors, or those might be sequestered.

RS-mediated regulation of TPP biosynthesis is adopted also by other commensal nasopharyngeal bacteria. *S. pneumoniae* is a Gram-positive firmicute, while *H. influenzae* is a Gram-negative gamma-proteobacterium, both distantly related to each other and to *Neisseria*. Using Riboswitch scanner, we investigated the genomes of *S. pneumoniae* (serotype 4, strain TIGR4, NC_003028.3) and *H. influenzae* (type B, strain 10,810, NC_016809.1), and identified four and three TPP RSs candidates, respectively (Table S2). These four pneumococcal candidates match the TPP RSs mapped in previous genomic investigations [37], whereas in *Haemophilus*, RS-mediated regulation remains largely unstudied, with the exception of *lysT* lysine RS [38]. Nevertheless, all three clinically relevant pathogens use several TPP RSs, with all four pneumococcal RSs predicted to regulate transcription, while all three from *H. influenzae* should control translation [27]. Further studies are required to validate the functionality of these putative RSs.

Research in the interplay among nasopharyngeal pathogens in scavenging nutrients such as thiamine within the nasopharynx remains a challenge as the microbiota varies between individuals. Our study here could pave the way for further investigations into this understudied field to better understand how nasopharyngeal bacteria colonize this niche and compete for nutrients.

## Materials and methods

### Bacterial strains and growth conditions

The strains, oligonucleotides and plasmids used in this study are listed in Table 1.

**Table 1.** Strains, oligonucleotides and plasmids used in this study.

Strain	Comments	Reference
*Neisseria meningitidis* MC58	Serogroup B, ATCC BAA-335	[46]
*Neisseria meningitidis* Z2491	Serogroup A	[47]
*Neisseria gonorrhoeae* MS11	MS11_mk_ P+	[48]
*Neisseria lactamica*	NCTC 10616	
*Escherichia coli* XL10-Gold		Agilent Technologies
*N. meningitidis* MC58 ΔRS		This study
*N. meningitidis* MC58 Δ*thiC*		This study
**Oligonucleotide**	**Description^a^**	**Sequence (5´-3´)**
**Probes for Northern blot**		
NMB2040_p1b	*thiC* probe A	CCCGATCGGGTGTAAAGGGTATTTCTCAGCCGCCTAAACATCAGGCAGC
NMB2040_p2b	*thiC* probe B	CAAAGGCACGCGGATGTCGTCGCGGCTGCCTTGCAGATACACGCGTTCC
16S_rRNA	16S rRNA probe	GACACGCGGCATGGCTGGATCAGGCTTGCGCCCATTGTCCAAAATTCCCC
**Primers used in the primer extension**		
thiC_PE_R1	Primer extension primer	GCAGAATCCGTGAAAACTCC
2040_P3_RV	rv PCR primer, used with thiC_MC58_cl_f	GGAGCGTTTTGCGTTTTCAGACG
**PCR primers to generate the inserts for the reporter plasmids^b^**		
thiC_MC58_cl_f	*N. meningitidis* MC58, fw	TTTGAATTCGCCGTCCGGCGGTTTATGCCTG
thiC_MC58_cl_r1	*N. meningitidis* MC58, rv	TTTCCCGGGCATAAAAAAATGCTCCTGTTTTCTCG
thiC_Z2491_cl_f	*N. meningitidis* Z2491, fw	TTTGAATTCGCCGTCCGTCGGTTTATGCCTG
thiC_MS11_cl_f	*N. gonorrhoeae* MS11, fw	TTTGAATTCTCCGTCCGGCGGCTTATGCCTG
thiC_Nl_cl_f	*N. lactamica*, fw	TTTGAATTCGTCCGAGGCCGGTTTATGTTTG
thiC_os_cl_r1	Z2491, MS11, *N. lactamica*, rv	TTTCCCGGGCATAAAAAATGCTCCTGTTTTCTCG
thiC_MC58_cl_r2	MC58, rv, used for the ΔRS	TTTCCCGGGGTCGGCAAGCTCGCGCGCTTC
thiC_RSKO_r1	MC58, rv, used for the ΔRS	AATGCTCCTGTTTTCTCGTTTAGAATCAATGTTAACCAAAATTAAATCAC
thiC_RSKO_f2	MC58, fw, used for the ΔRS	GTGATTTAATTTTGGTTAACATTGATTCTAAACGAGAAAACAGGAGCATT
cytX_EGFP_f	MC58 *cytX*, fw	TTTGAATTCCAGTACGGTGTTGCCTCGCCTTGCC
cytX_EGFP_r	MC58 *cytX*, rv	TTTCCCGGGGGCATTGCCCGACATATCAAGTCC
thiD_EGFP_f	MC58 *thiD*, fw	TTTGAATTCCAGTTGCTACGATGCACCCCGC
thiD_EGFP_r	MC58 *thiD*, rv	TTTCCCGGGGGTAAGCGTTTGCACAAAAGAGC
**QuikChange® PCR primers to site-directed mutagenesis^c^**		
thiC_qc_at_f	mutAT, fw	GATTTAATTTTGGTTAACATTGA**GGAC**GGGGTGCTGCCTGATGTTTAGGC
thiC_qc_at_r	mutAT, rv	GCCTAAACATCAGGCAGCACCCC**GTCC**TCAATGTTAACCAAAATTAAATC
thiC_qc_bs_f	mutBS, fw	GTGCTGCCTGATGTTTAGGCGGCT**CATC**AATACCCTTTACACCCGATCGG
thiC_qc_bs_r	mutBS, rv	CCGATCGGGTGTAAAGGGTATT**GATG**AGCCGCCTAAACATCAGGCAGCAC
thiC_qc_t_f	mutT, fw	AATGCCGTCTGAAAACGCAAAAC**AAAA**CTGTTTCTTTAATTCTAAACGAG
thiC_qc_t_r	mutT, rv	CTCGTTTAGAATTAAAGAAACAG**TTTT**GTTTTGCGTTTTCAGACGGCATT
**PCR primers to generate Δ*thiC*^d^**		
thiC_mut_5_f	5´ region, fw	*ATGCCGTCTGAAGAT*GAAGCAGCGTTTTATCCAAACCG
thiC_mut_5_r	5´ region, rv	TGGTGCAAGTCACACGAACACGAACAAATCAGGCATAAACCGCCGGACGG
thiC_mut_er_f	Erythromycin cassette, fw	CCGTCCGGCGGTTTATGCCTGATTTGTTCGTGTTCGTGTGACTTGCACCA
thiC_mut_er_r	Erythromycin cassette, rv	CGGCATTTTTTGCTTGACGTTTAACATGTTGCTGATTAAGACGAGCAATA
thiC_mut_3_f	3´ region, fw	TATTGCTCGTCTTAATCAGCAACATGTTAAACGTCAAGCAAAAAATGCCG
thiC_mut_3_r	3´ region, rv	*ATGCCGTCTGAACT*CGCTGTTTTCTGTTTCGCTGTTTTC
**PCR primers to generate ΔRS^d^**		
thiC_RSmut_5_r	5´ region, rv	TATTGCTCGTCTTAATCAGCAACATGATTTTTCGCGGAAGTAATGTTTG
thiC_RSmut_er_f	Erythromycin cassette, fw	CAAATCAGGCATAAACCGCCGGACGGTTCGTGTTCGTGTGACTTGCACCA
thiC_RSmut_er_r	Erythromycin cassette, rv	CAAACATTACTTCCGCGAAAAATCATGTTGCTGATTAAGACGAGCAATA
thiC_RSmut_RS_f	Mutated RS region, fw	TGGTGCAAGTCACACGAACACGAACCGTCCGGCGGTTTATGCCTGATTTG
thiC_RSmut_RS_r	Mutated RS region, rv	GTTTTGGCGGTTTTTTTTGGCGTAGTCATAAAAAAATGCTCCTGTTTTC
thiC_RSmut_3_f	3´ region, fw	GAAAACAGGAGCATTTTTTTATGACTACGCCAAAAAAAACCGCCAAAAC
thiC_RSmut_3_r	3´ region, rv	*ATGCCGTCTGAACT*GGCGGTCATCGGCACATAACGCAG
**Plasmid**	**Description**	**Reference**
pEGFP-N2	EGFP translational fusions reporter plasmid, Kan^R^	Clontech, [23]
pSp72/LytA-Erm	Template plasmid to obtain the erythromycin resistance cassette.	[49]
pRS	pEGFP-N2 with *thiC* promoter and 5ʹUTR +3 (ATG) from *N. meningitidis* MC58 fused with EGFP	This study
pΔRS	pRS, in which the 5ʹUTR is mutated with a deletion of the riboswitch element	This study
pRS(*N. men* A)	pEGFP-N2 with *thiC* promoter and 5ʹUTR +3 (ATG) from *N. meningitidis* Z2491 fused with EGFP	This study
pRS(*N. gon*)	pEGFP-N2 with *thiC* promoter and 5ʹUTR +3 (ATG) from *N. gonorrhoeae* MS11 fused with EGFP	This study
pRS(*N. lac*)	pEGFP-N2 with *thiC* promoter and 5ʹUTR +3 (ATG) from *N. lactamica* fused with EGFP	This study
pRS(mutAT)	pRS, in which the 5ʹUTR is mutated with AACA9-12GGAC, that disrupts the antiterminator structure.	This study
pRS(mutBS)	pRS, in which the 5ʹUTR is mutated with GAGA40-43CATC, that disrupts the ligand binding.	This study
pRS(mutT)	pRS, in which the 5ʹUTR is mutated with GCTC148-151AAAA, that disrupts the terminator structure.	This study
p*cytX*-EGFP	pEGFP-N2 with *cytX* promoter and 5ʹUTR +15 coding bp from *N. meningitidis* MC58 fused with EGFP	This study
p*thiD*-EGFP	pEGFP-N2 with *thiD* promoter and 5ʹUTR +30 coding bp from *N. meningitidis* MC58 fused with EGFP	This study

^a^Fw: forward; rv: reverse.

^b^SmaI (CCCGGG) and EcoRI (GAATTC) restriction sites are underlined.

^c^The mutated regions are in bold.

^d^The *Neisseria* uptake sequence is in italic.

*N. meningitidis* was grown in Brain Heart Infusion broth (BHI, Sigma-Aldrich, 37 g dissolved in 1 L dH_2_O) or on BHI agar (Sigma-Aldrich) (1.5% w/v) supplemented with 1 g/L starch (Sigma-Aldrich) and horse serum (Thermo Fisher Scientific). Bacteria on solid media were incubated overnight at 37°C with 5% CO_2_. Liquid cultures were inoculated to an initial optical density (OD) measured at 600 nm of ~0.05 and grown at 37°C with shaking (130 rpm) to the indicated OD_600_.

*E. coli* was grown in Luria-Bertani (LB) broth (2% w/v in dH_2_O, Sigma-Aldrich) or on LB agar (1% w/v) plates. Liquid cultures of *E. coli* were grown in 4 ml of media inoculated from a single colony overnight at 37°C with shaking (180 rpm). Overnight grown bacteria were diluted 1 in 100 in media and grown to an OD_600_ of ~0.5.

When necessary, antibiotics were added to the following final concentrations: kanamycin, 50 μg ml^−1^; erythromycin, 2.5 μg ml^−1^.

Spent medium was produced by growing *E. coli* or *N. meningitidis* to stationary phase in LB or BHI liquid media, respectively. After removal of the bacteria by centrifugation, the media were sterile filtered through a 0.45 µm filter.

Bacterial and cell lysates were produced by mechanical lysis with glass beads of *E. coli* MG1655, *N. meningitidis* MC58 or A459 human alveolar epithelial cells (ATCC CCL-185). Bacterial or human cells were collected after growth, washed with water and resuspended in 1 mL of milliQ water. The solutions were transferred to a Lysing Matrix B vial (MP Biomedicals) and processed in a Thermo Savant FastPrep FP120 Cell Homogenizer for 40 s at a setting of 6.0. The final volume was set with sterile water to 1/50 of the culturing volume.

In order to assess the effect of different thiamine sources on the TPP-mediated expression of *thiC* in *E. coli* reporter strains and *N. meningitidis*, the following experimental procedures were adopted. For *E. coli*, each strain was cultured in 40 mL fresh or spent LB medium with shaking until OD_600_ of 0.1. One milliliter sample was harvested before splitting the culture in 5 aliquots of 6 mL each. Except for the aliquot named ‘no suppl.’, each aliquot was supplemented with 120 µL of sterile dH_2_O, spent LB, *E. coli* lysate and thiamine water solution (final concentration of 500 µM). OD_600_ was measured at the indicated time points and 1 mL aliquot was harvested. The samples were prepared for Western blot as described later. In the case of the *N. meningitidis* experiment, each strain was cultured in 130 mL fresh or spent BHI medium until OD_600_ of 0.1. The cultures were split into 12 mL aliquots, without (‘no suppl.’) or with 240 µL of the following supplements: sterile dH_2_O, spent BHI, *N. meningitidis* lysate and thiamine water solution (final concentration of 1 mM). Ten milliliters aliquots were harvested and processed to isolate total RNA as described later.

### *Computational analysis of* thiC *riboswitch*

The online tool Riboswitch Scanner was utilized with default parameters to identify putative RS elements in the genomes of *N. meningitidis* MC58 (accession number: NC_003112.2), *Streptococcus pneumoniae* (serotype 4, strain TIGR4, NC_003028.3) and *Haemophilus influenzae* (type B, strain 10810, NC_016809.1) [26].

The retrieval of orthologous neisserial regions was performed with NCBI BLAST and multiple sequence alignments and analysis of conservation with Clustal Omega [39]. Sequence alignments were visualized with Jalview [40].

The secondary structures of the two conformations of the aptamer and expression platform of *thiC* RS were, predicted combining information from published validated structures of TPP RSs [27 28,] with computational analysis with RNAfold (v2.4) [41]. RNA structures were visualized with VARNA (v3-93) [42].

### Reporter plasmid construction

All plasmids used in this study are listed in Table 1. Recombinant DNA work was performed according to standard protocols [43]. The correct nucleotide sequences of all constructs were confirmed by automated sequencing (Eurofins Genomics, Martinsried, Germany).

The reporter plasmids were constructed by inserting the region containing the *thiC* promoter, 5´UTR and the translational start codon in a translational fusion to EGFP gene in the plasmid pEGFP-N2. The corresponding regions from *N. meningitidis* MC58, *N. meningitidis* Z2491, *N. gonorrhoeae* MS11 and *N. lactamica* were amplified by PCR from genomic DNA of the respective strains with the primers indicated in Table 1. The inserts and the plasmid were digested with restriction enzymes SmaI and EcoRI (New England Biolabs), according to the manufacturer’s protocols. Ligation of the inserts with pEGFP-N2 was performed with T4 DNA ligase (Thermo Fisher Scientific), according to manufacturer’s protocol.

In order to obtain a functional 5´UTR that lacks the RS element (ΔRS), the region containing the promoter, transcriptional start site and first 7 transcribed bp was amplified by PCR with primers thic_MC_c1_f and PR thiC_RSKO_r1. A second region including the last 31 bp of the 5´UTR (to maintain the ribosome binding site) and the first 60 coding bp was amplified with primers thic_RSKO_f2 and PR thiC_MC58_cl_r2. The two products were ligated with the Gibson Assembly® system (New England Biolabs). The final DNA insert was amplified by PCR from the ligation product using primers thic_MC_c1_f and thiC_MC58_cl_r1. The resulting product contains the *thiC* promoter, a shortened 5´UTR lacking the RS aptamer but maintaining the ribosome binding site and the translational start codon. The insert was digested with the restriction enzymes SmaI and EcoRI and ligated to digested pEGFP-N2 to obtain pΔRS.

In order to obtain mutated versions of *thiC* RS element, site-directed mutagenesis was performed according to the instruction manual of the QuikChange® mutagenesis kit. The mutAT mutant was generated with oligonucleotides thiC_qc_at_f and thiC_qc_at_r; the mutBS mutant with oligonucleotides thiC_qc_bs_f and thiC_qc_bs_r; mutT with oligonucleotides thiC_qc_t_f and thiC_qc_t_r.

All reporter plasmids were transformed into *E. coli* XL10-Gold® (Agilent Technologies), according to manufacturer’s manual.

### *Construction of* N. meningitidis *mutants*

Genomic DNA from bacterial strains used in this study was isolated using the Wizard® Genomic DNA Purification Kit (Promega), according to manufacturer’s protocol. The method used to obtain chromosomal mutants in *N. meningitidis* is based on its natural uptake of foreign DNA and homologous recombination. Each construct for the transformation is designed to have ~1 kbp of homologous 5´ region, followed by an antibiotic resistance cassette and a~1 kbp 3´ homologous region. In this case, the homologous recombination will replace the region between the two homologous segments with the resistance cassette.

In order to obtain the Δ*thiC N. meningitidis* MC58 mutant, the 5´ region was amplified by PCR from genomic DNA with primers thiC_mut_5_f and thiC_mut_5_r, the 3´ region with primers thiC_mut_3_f and thiC_mut_3_r, and the erythromycin cassette from pSp72/LytA-Erm with primers thiC_mut_er_f and thiC_mut_er_r. Complementary tails allow ligation using the Gibson Assembly® system (New England Biolabs) for *in vitro* ligation. The ligated product was then amplified by PCR with primers thiC_mut_5_f and thiC_mut_3_r. Primers thiC_mut_5_f and thiC_mut_3_r have a tail with the *Neisseria* uptake sequence, to enhance the uptake of the DNA fragment by the bacteria [44].

To generate the ΔRS mutant, the region containing the promoter and 5´UTR of *thiC* was deleted and replaced by the corresponding region lacking the RS element, as constructed in the reporter plasmid pΔRS. The 5´ region was amplified by PCR from genomic DNA with primers thiC_mut_5_f and thiC_RSmut_5_r, the 3´ region with primers thiC_RSmut_3_f and thiC_RSmut_3_r, the erythromycin cassette from pSp72/LytA-Erm with primers thiC_RSmut_er_f and thiC_RSmut_er_r, the mutated ΔRS region to replace from pΔRS with primers thiC_RSmut_RS_f and thiC_RSmut_RS_r. *In vitro* ligation of the four fragments was performed using the Gibson Assembly® kit.

In order to transform *N. meningitidis*, 5 µL of bacterial suspension were seeded in a ~ 3 cm diameter region of a BHI plate and allowed to dry. Five microliters (~100–500 ng) of DNA fragment were spread on top of the bacteria. The plate was incubated 3 h at 37°C with 5% CO_2_. The grown bacteria were then collected and spread on a fresh selective (erythromycin, 2.5 μg ml^−1^) plate and incubated overnight. Resistant colonies were picked, the presence of the cassette was checked by colony PCR and the correctness of the construct was verified by sequencing.

### RNA isolation and northern blot analysis

*N. meningitidis* was grown in aerated liquid culture at 120 rpm, to the indicated OD_600_, and RNA was isolated using the RNeasy Midiprep Kit (Qiagen) following the manufacturer’s protocol. The purity and integrity of RNA were determined by spectrophotometry.

For Northern blotting, 20 μg of total RNA was separated on an agarose/formaldehyde gel as previously described [45]. RNA was then transferred to Hybond N+ membranes (GE Healthcare Life Sciences) by capillarity blotting. The membranes were hybridized overnight with ^32^P ɣ-labelled DNA oligonucleotides. Northern blots were developed using a Biorad Molecular Imager® FX. Probes for detecting *thiC* transcripts were designed in the 5´UTR (probe A) and in the coding region (probe B) and are listed in Table 1.

### Primer extension

Primer extension was performed using the High Capacity cDNA Reverse Transcription Kit (Applied Biosystems), with a modified protocol. Two micrograms of total RNA isolated from *N. meningitidis* MC58 wt and ΔRS, grown in BHI to exponential phase were used as template for the primer extension reaction. 13.2 µL of RNA were mixed with 2 µL 10x RT buffer, 1 µL RNase inhibitor and 2 µL ^32^P ɣ-labelled DNA oligonucleotide thiC_PE_R1. A third sample contains no RNA, as a negative control. The annealing step was performed by heating the mixtures to 85°C for 5 min, followed by slowly cooling down to 25°C in 20 min. 0.8 µL of 100 mM dNTPs mix and 1 µL MultiScribe^TM^ Reverse Transcriptase were added. The samples were then incubated 2 h at 37°C for the reverse transcription reaction. Finally, 20 µL of formamide 2x RNA loading dye (New England Biolabs) were added to the products. Products were denatured at 80°C for 3 min in formamide sample buffer. A PCR product of the *thiC* promoter region and 5´UTR was generated using primers thiC_MC58_cl_f (used also for cloning) and 2040_P3_RV, and sequencing was performed using the labelled thiC_PE_R1 primer according to standard methods [43]. The samples were heated at 95°C for 10 min and separated by electrophoresis on denaturing 8% polyacrylamide/urea gel. The gel was dried and radioactive signals detected as previously described.

### Western blot analysis

For immunoblotting, bacterial pellet was spun down at 7,000 g for 10 min and lysed in Buffer A (200 mM KCl, 50 mM Tris-HCl pH 8.0, 1 mM EDTA, 10% Glycerol) at 98°C. Cell debris was spun down at 12,000 g for 5 min and lysate was collected. 4x SDS-loading buffer was added to a normalized volume of 400 μL/OD_600_ and boiled at 98°C for 10 min before 8 μL protein samples were subjected to electrophoresis on 4–12% Bis-Tris gel (Thermo Fisher Scientific) and run at 110 V. Gels were transferred to PVDF membranes using the Trans-Blot Turbo system (Bio-Rad). Membranes were blocked in 5% milk for 1 h at room temperature (RT) and incubated with primary antibody in PBS + 0.1% Tween, for 1 h at RT. After washing membranes three times for 5 min, secondary antibody was added in PBS + 0.1% Tween for 1 h. Membranes were washed three times for 5 min, treated with Amersham ECL reagents (GE Healthcare Life Sciences) and visualized by GelDoc XRS+ (Bio-Rad). Quantification of the signals was obtained with the software ImageJ (v1.52 h, NIH, USA). Each experiment was performed in at least a triplicate and each blot displayed is a representative result.

Primary antibodies used were anti-RecA (Abcam, ab63797) and anti-GFP (Clontech Monoclonal Ab JL8, 632381). Secondary antibodies were anti-rabbit IgG-HRP (GE Healthcare Life Sciences, RNP4301) for anti-RecA and anti-mouse IgG-HRP (GE Healthcare Life Sciences, NXA931) for anti-GFP.
