# Supplementary material for: Characterization of a transcriptional TPP riboswitch in the human pathogen Neisseria meningitidis
Source: RNA Biol. 2020 Feb 20;17(5):718–30. doi: 10.1080/15476286.2020.1727188 (PMC7237195; doi:10.1080/15476286.2020.1727188)
Supplement: Supplemental Material [file krnb-17-05-1727188-s001.docx]

# **Characterisation of a transcriptional TPP riboswitch in the human pathogen *Neisseria meningitidis***

# **Supplementary material**

**Francesco Righetti,^a^ Solange Lise Materne,^a^ John Boss,^a^ Hannes Eichner,^a^ Emmanuelle Charpentier,^b,c,d,e,f,^ and Edmund Loh*^a,g^**

**^a^**Department of Microbiology, Tumor- and Cell biology, BioClinicum, Karolinska University Hospital, 171 64 Stockholm, Sweden

**^b^**Max Planck Unit for the Science of Pathogens, Berlin, Germany

**^c^**Department of Regulation in Infection Biology, Max Planck Institute for Infection Biology, Berlin, Germany

**^d^**Institute for Biology, Humboldt University, Berlin, Germany

**^e^**Department of Regulation in Infection Biology, Helmholtz Centre for Infection Research, Braunschweig, Germany

**^f^**The Laboratory for Molecular Infection Medicine Sweden (MIMS), Umeå Centre for Microbial Research (UCMR), Department of Molecular Biology, Umeå University, Umeå, Sweden

**^g^**SCELSE, Nanyang Technological University, 639798, Singapore

*Corresponding author

E-mail: edmund.loh@ki.se

## **Table S1**

| **Family** | **Gene** | **Product** | **E-value** |
| --- | --- | --- | --- |
| preQ1 | NMB0317 | 7-cyano-7-deazaguanine reductase | 5e-10 |
| SAM I-IV | *metK* | S-adenosylmethionine synthetase | 3e-05 |
| TPP | *cytX* | hydroxymethylpyrimidine transporter | 5.4e-10 |
| TPP | *thiC* | phosphomethylpyrimidine synthase | 1.1e-08 |

**Table S1. Riboswitch Scanner output for *N. meningitidis* MC58 genome.**

The four RS candidates identified by Riboswitch Scanner are listed. RS family, gene names, annotated gene product and estimated E-value of the RS element are indicated.

## **Figure S1**


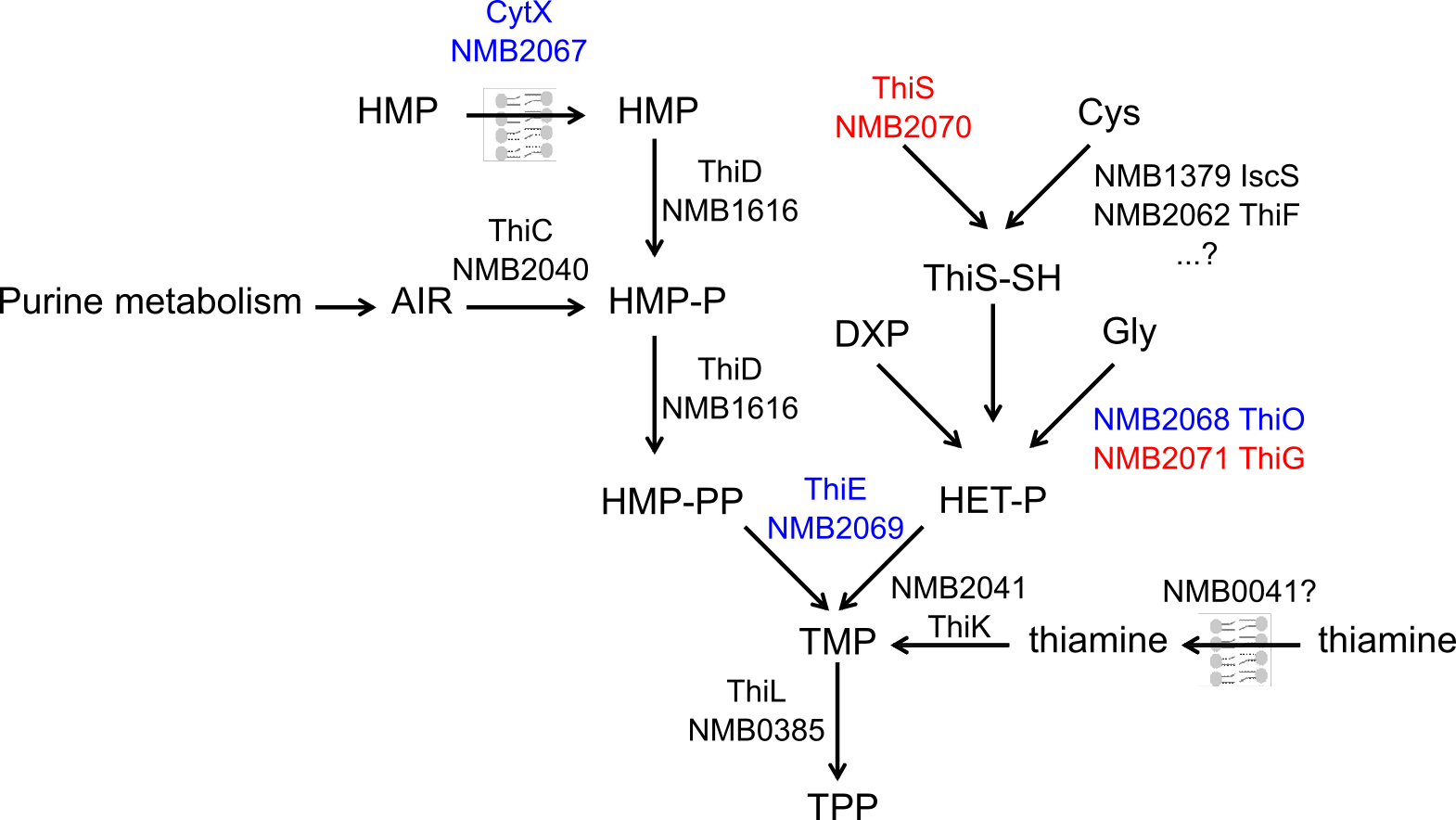


**Figure S1. Simplified schematic representation of the TPP metabolism in *N. meningitidis*.**

The expected TPP metabolism in *N. meningitidis* was reconstructed based on genomic data. HMP: hydroxymethylpyrimidine (-P and -PP: mono- and di-phosphorylated); AIR: phosphoaminoimidazole riboside; Cys: L-cysteine; DXP: 1-deoxy-D-xylulose phosphate; Gly: glycine; HET-P: hydroxyethylthiazole phosphate; TMP and TPP: thiamine mono- and pyro-phosphate. Per each step, the names of the enzymes and transporters are indicated, together with their gene ID from *N. meningitidis* MC58 genome annotation. Genes putatively located within the same transcriptional unit are marked with the same color (blue or red).

## **Figure S2**

**
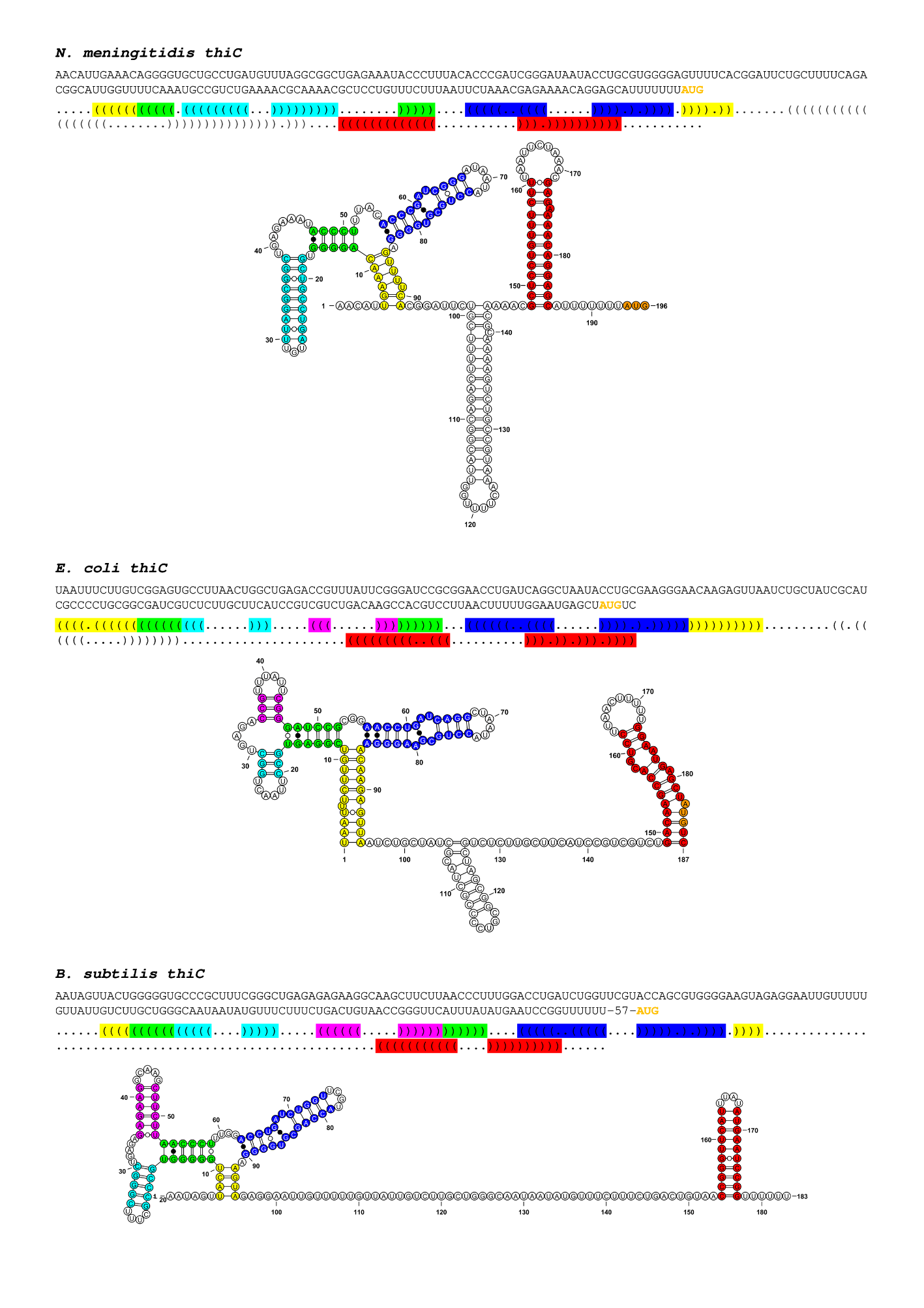
**

**Figure S2. Structural comparison of *N. meningitidis*, *E. coli* and *B. subtilis thiC* TPP RSs.**

The structures are indicated in Vienna dots and brackets format, as well as in a secondary structure drawing produced with VARNA applet. Conserved structural elements (stems) are labelled with same colors, the AUG start codon is depicted in orange.

## **Table S2**

| Species | Downstream gene | Gene product | Putative mechanism |
| --- | --- | --- | --- |
| *S. pneumoniae* | SP_0716 / *tenA* | Aminopyrimidine aminohydrolase | transcriptional |
| *S. pneumoniae* | SP_0719 / *ykoE* | Thiamine permease | transcriptional |
| *S. pneumoniae* | SP_0726 / *thiD* | Hydroxymethylpyrimidine kinase | transcriptional |
| *S. pneumoniae* | SP_2199 / *thiX* | Hydroxymethylpyrimidine transporter | transcriptional |
| *H. influenzae* | HIB_04670 / *thiZ* | Hydroxymethylpyrimidine transporter | translational |
| *H. influenzae* | HIB_05260 / *thiM* | Hydroxyethylthiazole kinase | translational |
| *H. influenzae* | HIB_11890 / *thiB* | Thiamine transporter | translational |

**Table S2. TPP RSs predicted in the genomes of the human nasopharyngeal bacteria *Streptococcus pneumoniae* and *Haemophilus influenzae*.**

The TPP RS candidates identified by Riboswitch Scanner in *S. pneumoniae* (serotype 4, strain TIGR4, NC_003028.3) and *H. influenzae* (type B, strain 10810, NC_016809.1) are listed. Downstream gene names and ID, annotated gene products and predicted regulatory mechanisms are indicated. *S. pneumoniae* *thiD* TPP RS was not detected in [Rodionov *et al.* (2002)](#_ENREF_1).

# **References**

Rodionov DA, Vitreschak AG, Mironov AA, and Gelfand MS (2002). Comparative genomics of thiamin biosynthesis in procaryotes. New genes and regulatory mechanisms. *J Biol Chem* 277**,** 48949-48959. doi: 10.1074/jbc.M208965200.
